# NOD2-C2 - a novel NOD2 isoform activating NF-κB in a muramyl dipeptide-independent manner

**DOI:** 10.1186/1756-0500-3-224

**Published:** 2010-08-10

**Authors:** Marcel Kramer, Janne Boeck, Daniela Reichenbach, Christoph Kaether, Stefan Schreiber, Matthias Platzer, Philip Rosenstiel, Klaus Huse

**Affiliations:** 1 Genome Analysis, Leibniz Institute for Age Research - Fritz Lipmann Institute, Beutenbergstrasse 11, 07745 Jena, Germany; 2 Membrane Trafficking, Leibniz Institute for Age Research - Fritz Lipmann Institute, Beutenbergstrasse 11, 07745 Jena, Germany; 3 Institute of Clinical Molecular Biology, University Hospital Schleswig-Holstein, Christian Albrechts University, Schittenhelmstrasse 12, 24105 Kiel, Germany

## Abstract

**Background:**

The innate immune system employs several receptor families that form the basis of sensing pathogen-associated molecular patterns. NOD (nucleotide-binding and oligomerization domain) like receptors (NLRs) comprise a group of cytosolic proteins that trigger protective responses upon recognition of intracellular danger signals. NOD2 displays a tandem caspase recruitment domain (CARD) architecture, which is unique within the NLR family.

**Findings:**

Here, we report a novel alternative transcript of the *NOD2 *gene, which codes for a truncated tandem CARD only protein, called NOD2-C2. The transcript isoform is highest expressed in leucocytes, a natural barrier against pathogen invasion, and is strictly linked to promoter usage as well as predominantly to one allele of the single nucleotide polymorphism rs2067085. Contrary to a previously identified truncated single CARD NOD2 isoform, NOD2-S, NOD2-C2 is able to activate NF-κB in a dose dependent manner independently of muramyl dipeptide (MDP). On the other hand NOD2-C2 competes with MDPs ability to activate the NOD2-driven NF-κB signaling cascade.

**Conclusion:**

NOD2 transcripts having included an alternative exon downstream of exon 3 (exon 3a) are the endogenous equivalents of a previously described *in vitro *construct with the putative protein composed of only the two N-terminal CARDs. This protein form (NOD2-C2) activates NF-κB independent of an MDP stimulus and is a potential regulator of NOD2 signaling.

## Background

The innate immune system uses several molecules that sense pathogen-associated molecular patterns (PAMPs) including Toll-like, RIG-1 (retinoic acid inducible gene protein 1)-like and the NOD (nucleotide-binding and oligomerization domain)-like receptors (NLRs) to trigger a protective response against intracellular danger signals, e.g. cytoinvasive pathogens. The NLRs family consists of more than 20 related members defined by a tripartite structure consisting of: (i) a variable N-terminal protein-protein interaction domain, defined by the caspase recruitment domain (CARD), pyrin domain (PYD), or the baculovirus inhibitor domain (BIR); (ii) a centrally located NOD domain facilitating self-oligomerization during activation [1], and (iii) a C-terminal leucine-rich repeat (LRR) responsible for binding/detecting of PAMPs. The N-terminal effector binding domains are essential elements of the NLRs to elicit a signal subsequent to NLR activation. In case of NOD1 and NOD2 (CARD15), the N-terminal domain is formed by a single or a tandem CARD, respectively. Both are pivotal for the induction of pro-inflammatory pathways in response to bacteria by inducing signaling pathways via NF-κB (nuclear factor kappa B) or MAPK (mitogen-activated protein kinases) and recognize bacterial cell wall components derived from peptidoglycan (PGN). NOD1 activity is triggered by γ-D-glutamyl-*meso*-diaminopimelic acid (*meso*-DAP) [2,3]. In contrast, NOD2 is activated by muramyl dipeptide (MDP), a peptidogylcan motif present in all Gram-positive and -negative bacteria [4,5]. Initial biochemical characterization of NOD1/2 revealed that both proteins induce NF-κB activation in a Toll-like receptor-independent fashion [6]. Upon ligand recognition, NOD1/2 undergo conformational changes and self-oligomerization. This process is followed by recruitment and activation of the serine threonine kinase RIP2 (receptor interacting protein 2) via homophilic CARD/CARD interaction, which is essential for the activation of NF-κB and MAPKs [7-9].

Despite insufficient expression of endogenous proteins hampering their analysis, *in vitro *enforced protein expression of NLRs has shed some light on the intracellular distribution of these molecules. In addition to their preponderant cytosolic localization, NOD1/2 were also found to be associated with the plasma membrane [10-14]. The plasma membrane association of NOD2 has been linked to activation of downstream signaling events since point-mutations of NOD2 interfering with their membrane association capacity resulted in blunted NOD2-mediated NF-κB activation [10]. However, the latter studies are difficult to interpret as the same mutations also interfered with MDP recognition [12]. These genetic variants in *NOD2 *have been associated with susceptibility for Crohn's disease (CD), a chronic inflammatory bowel disorder. Three major CD-associated polymorphisms (Arg702Trp, Gly908Arg and Leu1007sinsC) are located in or near the LRR domain and are associated with a decreased NOD2 activation by MDP [15-18]. Moreover, a recent genome-wide association study of Leprosy identified NOD2 sequence variants to be significantly associated with affected status [19].

It has been shown that proinflammatory stimuli, such as TNF-α, IFN-γ and lipopolysaccharide (LPS) activate *NOD2 *gene expression in intestinal epithelial cell lines and primary intestinal epithelial cells as well as monocytic HL-60 cells [20,21]. This up-regulation is at least in part dependent on the binding of NF-κB to a proximal κB-binding element (-26) of a *NOD2 *promoter region in front of the canonical first exon. Rosenstiel and colleagues [22] identified two novel exons of the *NOD2 *gene (designated exon 1a and 1b), which are spliced to the canonical exon 2 and constitute the 5'-untranslated region of two alternative transcripts (i.e. exon 1a/1b/2 and exon 1a/2). These transcript isoforms are abundantly expressed and seem to comprise the majority of NOD2 transcripts under physiological conditions. Further studies on NOD2 mRNA splicing revealed a short isoform, NOD2-S, caused by skipping of the third exon and a subsequent premature stop codon in exon 4 [22,23]. The truncated protein is restricted to only one of its unique tandem CARD domains and has been shown to be an endogenous inhibitor of the NOD2/receptor-interacting protein kinase 2 (RIP2)-induced signaling pathway [22]. NOD2-S competes with the downstream target of NOD2, RIP2, for binding of NOD2. This inhibits the signaling cascade within the NOD2-induced activation of the NF-κB driven immune response. NOD2-S induced expression and secretion of the pro-inflammatory cytokine IL-8 and NOD2/RIP2-dependent IL-1β maturation. This suggests an important role of the CARD-only containing isoform in the modulation of NOD2/RIP2 signaling events [22]. In contrast, Leung *et al. *and Ogura *et al. *reported that an artificial truncated isoform of NOD2 comprising both CARDs is able to activate NF-κB independent of a MDP stimulation [23,24]. It is still an unresolved issue why NOD2 exhibits a tandem CARD motif. However, our results indicate a differential role of single and tandem CARD domains in NLRs.

In this report, we present an endogenous *NOD2 *alternative transcript coding for a protein containing the two CARDs only, NOD2-C2. We identified this splicing event to be both promoter as well as single nucleotide polymorphism (SNP) dependent and initially verified functional consequences with respect to the divergent roles of single and tandem CARD isoforms in NF-κB-induced inflammatory immune response.

## Material and methods

### Cell culture

Human EBV transformed lymphoblastoid cell lines (LCLs) GM10847, GM10854, GM10854, GM12760, GM12864, GM15215, GM15324, GM15386, GM18502, GM18552, GM18858, GM18972, GM19140, and GM19204 were obtained from the Coriell Cell Repository (Camden, USA). C0766 and the chimpanzee cell line EB176(JC) were from European Collection of Cell Cultures (ECACC, Salisbury, UK). Cells were cultured at 37°C in RPMI 1640 medium (Gibco, Eggenstein, Germany) supplemented with 15% FCS (Gibco) and 2 mM L-Glutamine (Gibco) in a 5% CO_2 _atmosphere and 95% humidity.

### Nucleic acids

Genomic DNA was isolated from the indicated cell lines using the Blood & Cell Culture DNA Mini Kit (Qiagen, Hilden, Germany) according to standard protocols. Total RNA was isolated from the cell lines using the RNeasy Mini Kit (Qiagen, Hilden, Germany) at three subsequent days according to manufacturer's protocol. cDNA first strand synthesis was performed with "Sprint RT Complete-Random Hexamer" cDNA synthesis kit (Clontech-Takara Bio Europe, Saint-Germain-en-Laye, France) following the manufacturer's recommendations. 5 μg total RNA were used for reverse transcription. Human tissue cDNAs were from Clontech (MTC Multiple Tissue cDNA Panels).

### PCR amplification and sequence analysis

PCR was performed with "BioMix white" (Bioline, Randolph, USA) according to manufacturer's protocol in a total volume of 25 μl including 3 μl cDNA from cell lines or 1 μl from human cDNA tissue panels (Clontech) and 10 pmol primer. In general, PCR reaction started with an initial denaturation at 93°C for 1 min, followed by 5 cycles of 30 sec denaturation (95°C), 30 sec annealing temperature temperature 1 and 1 min elongation at 72°C. Additional 25 cycles were performed with annealing temperature 2 followed by a final elongation of 20 min at 72°C. Primers and corresponding annealing temperatures 1/2 were as follows: quantNOD2ex24.f (5'-Fam-ATTGTCAGGAGGCTCCACAG-3') and quantNOD2ex24.r (5'-TGTCCGCATCGTCATTGAG-3') at 57°C/59°C, NOD2ex24.f (5'-ATTGTCAGGAGGCTCCACAG-3') and NOD2ex24.r (5'-TGTCCGCATCGTCATTGAG-3') at 57°C/59°C, NOD2ex23.f (5'- ATTGTCAGGAGGCTCCACAG-3') and NOD2ex23.r (5'-TAGAAGGAAGGCAGCCAATC-3') at 56°C/58°C, NOD2ex23a.f (5'-GGTACTTGGGCCTGTCAGAA-3') and NOD2ex23a.r (5'-AAACCTGGGTCCACCATACA-3') at 55°C/57°C, NOD2ex3a4.f (5'-TGTATGGTGGACCCAGGTTT-3') and NOD2ex3a4.r (5'-TGTCCGCATCGTCATTGAG-3') at 56°C/58°C, GAPDH.f (5'-AGGGCTGCTTTTAACTC-3') and GAPDH.r (5'-GCTTCACCACCTTCTTG-3') at 53°C/55°C as well as rs2067085.f (5'-CAGCCATGTGGAGAACATGC-3') and rs2067085.r (5'-GTTCATGGTGGTACAGCAGC-3') at 53°C/55°C. PCR products of the *NOD2 *locus were sequence analyzed using the ABI3730xl platform (Applied Biosystems, Foster City, USA).

To analyse the 5'-end of *NOD2 *exon 3a specific transcripts we performed 5'-rapid amplification of cDNA ends (RACE) on human leukocyte cDNA. Nested PCR was performed with "BioMix white" (Bioline) according to manufacturer's protocol in a total volume of 50 μl including 3 μl human leukocyte Marathon^® ^ready cDNA (Clontech) and 10 pmol primer. Marathon^® ^ready cDNA specific primers (AP1, AP2) were purchased from Clontech. Gene specific primers (GSP) were from Metabion (Martinsried, Germany). In general, PCR reaction started with an initial denaturation at 93°C for 1 min, followed by 5 cycles of 30 sec denaturation (95°C), 30 sec annealing temperature 1 and 90 sec elongation at 72°C. Additional 30 cycles were performed with annealing temperature 2 followed by a final elongation of 20 min at 72°C. Nested PCR was performed analogous using 3 μl of PCR product as template. Primers and corresponding annealing temperatures (1/2) were as follows: GSP1 (5'-AAACCTGGGTCCACCATACA-3', 55°C/57°C) and AP1 (Marathon^® ^cDNA amplification Kit, Clontech) as well as GSP2 (5'-CCATTTAGCCTTTCTGGGCCTCAGTTCTC-3', 65°C/67°C) and AP2 (Marathon^® ^cDNA amplification Kit, Clontech). PCR amplicons were cloned into pCR2.1TOPO using Topo TA Cloning^® ^Kit (Invitrogen, Karlsruhe, Germany) and subsequently sequenced.

### Capillary electrophoresis - laser induced fluorescence analysis

PCR amplification was carried out with 5'-6-carboxyfluorescein (FAM)-labeled forward or reverse primers (quantNOD2ex24.f/r). The FAM-labeled PCR products were appropriately diluted (up to 1/40) and 1 μl of the diluted sample was supplemented with 10 μl formamide (Roth, Karlsruhe, Germany) and 0.5 μl of GeneScan ROX 500 (Applied Biosystems) size standard. The mixture was denatured at 94°C for 3 min, and subsequently cooled on ice. The denatured products were then separated on an ABI3730xl capillary sequencer and analyzed using Gene Mapper 4.0 software (Applied Biosystems). The amount of each isoform within a PCR reaction was calculated by the area under the curve. *NOD2*-C2 percent fraction was calculated as exon 3 and 3a inclusion versus the sum of all transcripts spanning the exons 2-4. All values are given as mean ± standard deviation of PCRs on three independent RNA isolations.

### NOD2-C2 expression construct

The N-terminal FLAG-tagged NOD2-C2 expression construct pcDNA3.1-NOD2-C2 was obtained by overlapping extension PCR and cloned into the pcDNA3.1(+) (Invitrogen, Karlsruhe, Germany) expression plasmid. Therefore, four PCR products were amplified under standard PCR conditions using the following primers: NOD2ex1EcoRI.f3 (5'-GAATTCAGGAGGAAAGAGCAAGTGTC-3') and NOD2ex2ext.r (5'-CTTGTAACCTTGATACCAACCATTTCACAACCCGGAGAAT-3'), HBBin1.f (5'-GTTGGTATCAAGGTTACAAG-3') and HBBin1FLAG.rn (5'-CTTATCATCATCATCCTTGTAATCCTAAGGGTGGGAAAATAGAC-3'), NOD2ex2ext.fn (5'-ATTACAAGGATGATGATGATAAGTGCTCGCAGGAGGCTTTTCA-3') and NOD2ex3.r2 (5'-CCAATGGGACTGGTAATTCC-3') as well as NOD2in3df (5'-AGGCTGCTTGATCTTGCCAC-3') and NOD2ex3aEcoRI.r (5'-GAATTCAACCTGGGTCCACCATAC-3'). *NOD2 *exons 1-2 and 2-3 were amplified from cDNA of EBV-transformed human B-lymphocyte cells, *NOD2 *exons 3-3a from human testis cDNA (human MTC cDNA panel from Clontech) and HBB intron 1 from human genomic DNA (human genomic DNA pool from Roche, Mannheim, Germany). PCR products were subcloned with Topo TA Cloning^® ^(Invitrogen). Linker PCR was performed with *Pfu *Turbo Polymerase (Stratagen) and the cloned PCR fragments. Primers were used as previously listed, except HBBin1ext.f (5'-ATTCTCCGGGTTGTGAAATGGTTGGTATCAAGGTTACAAG-3') instead of HBBin1.f and HBBin1FLAGext.rn (5'-TGAAAAGCCTCCTGCGAGCACTTATCATCATCATCCTTGTAATC-3') instead of HBBin1FLAG.rn. Overlapping extension PCR was performed after addition of extra *Pfu *Turbo Polymerase and dNTPs to the mixture of all 4 linker PCR products. The overlapping extension PCR product conduced as template for an amplification of the whole NOD2-C2 expression construct with flanking primers (NOD2ex1EcoRI.f3 and NOD2ex3aEcoRI.r) using "BioMix white" (Bioline) according to manufacturer's protocol. PCR products were again subcloned with Topo TA Cloning^® ^(Invitrogen) and subsequently cloned into pcDNA3.1(+) with EcoRI and T4-DNA ligase (both from Fermentas, St. Leon-Rot, Germany) according to the manufacturer's instructions. All clones and constructs were sequence-verified with ABI3730xl.

### NF-κB activation assay

For quantification of NF-κB activity, transient transfection was performed using Fugene 6™ (Roche) according to the manufacturer's manual. One day before transfection human embryonal kidney HEK293 cells (ACC305, German Collection of Microorganisms and Cell Cultures, DSMZ Braunschweig, Germany) were seeded at a density of 2 × 10^4^/100 μl on a 96-well plate and cultured in Dulbecco's MEM medium (PAA Laboratories, Paschberg, Austria) supplemented with 10% fetal calf serum (FCS). Conditions for growth were 5% CO_2 _atmosphere and 95% humidity at 37°C. At the following day cells were cotransfected with pcDNA3.1empty vector (mock-control) and pcDNA3.1-NOD2 (a kind gift from G. Nunez, University of Michigan, Ann Arbor) or pcDNA3.1-NOD2-C2 together with 15 ng 3X NF-κB-luciferase reporter plasmid (Clontech) and 5 ng pRL-TK renilla reporter plasmid (Promega, Mannheim, Germany). For normalization of transfection efficiency and cell viability increasing concentrations of pcDNA3.1-NOD2-C2 or pcDNA3.1-NOD2 and pcDNA3.1 empty vector, respectively, up to a final concentration of 50 ng of DNA per well were used. 24 h after transfection, cells were stimulated with LD-MDP (10 μg/ml, Bacham) for 18 h. After incubation cells were washed in PBS and lysed in 1X Passive Lysis Buffer (Promega). Dual Luciferase Assay (DLA) was performed using the Dual Luciferase^® ^reporter assay system (Promega) and a MicroLumatPlus Luminometer (Berthold, Bad Wildbad, Germany). Each experiment was repeated independently three times. The results for firefly luciferase activity were normalized using the reference plasmid and expressed as relative light units (RLU).

### Immunofluorescence confocal-like microscopy

HEK293E and african green monkey kidney COS7 cells (ATCC, Rockville, MD, USA) were seeded on sterile poly-L-lysine coated coverslips and cultured in Dulbecco's MEM medium (PAA Laboratories, Paschberg, Austria) supplemented with 10% fetal calf serum (FCS) at 5% CO2 atmosphere, 95% humidity and 37°C. Cells were transfected after 24 h with Flag-NOD2-C2 using Lipofectamine 2000 (Invitrogen) according to manufacturer's instructions. After 48 h, cells were fixed and stained according to a standardized protocol [25]. As primary and secondary antibodies M2-anti-FLAG monoclonal mouse (F1804, Sigma-Aldrich, St. Louis, United States) and Alexa Fluor 555 goat anti-mouse IgG (A21424) from Invitrogen were used, respectively Confocal-like microscopy was performed on the Axiovert 200 microscopy system (Carl Zeiss, Jena, Germany).

### Statistical analysis

Statistical significance (p-value) was calculated with Sigma Plot 11.0 (Systat Software, Chicago, USA). Comparison of different groups of the NF-κB activation assay was performed with the "All Pairwise Multiple Comparison Procedure" (Tukey Test). For comparing unstimulated and MDP treated cells as well as for expression data the Student's t-test (t-test) was employed. In case where the normality test (Shapiro Wilks) failed, the Mann-Whitney Rank Sum Test (MWRST) was applied.

## Results

### Identification and expression patterns of *NOD2*-C2

**Figure 1:**
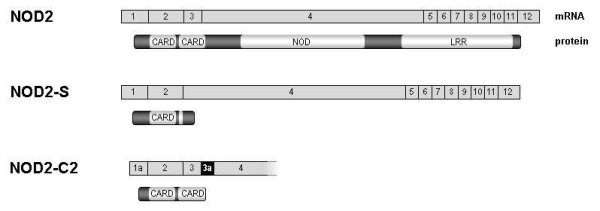
**Schematic transcript and protein representation of NOD2 and its isoforms NOD2-S as well as NOD2-C2**. The novel *NOD2*-C2 transcript contains an additional exon 3a highlighted in black. Contrary to NOD2-S, NOD2-C2 has a reconstructed second CARD domain, which differs in the last 9 amino acid compared to exon 3-4 spliced NOD2.

**Figure 2:**
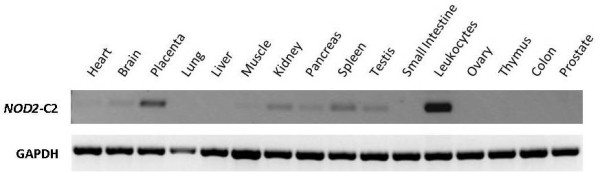
**Expression pattern of *NOD2*-C2 transcripts in human tissues**. RT-PCR specific for *NOD2 *exon 3a inclusion and *GAPDH *was performed on human cDNA tissue panels.

**Figure 3:**
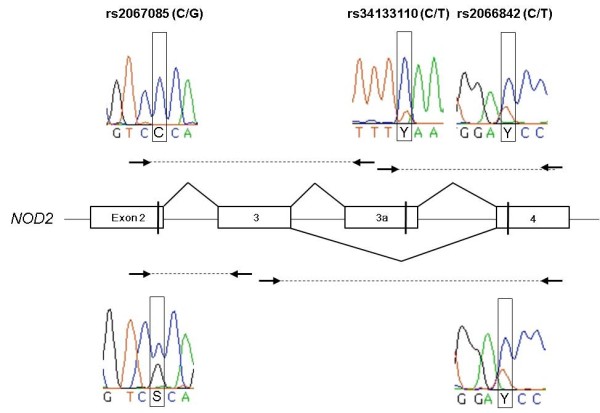
**Predominant inclusion of *NOD2 *exon 3a from the rs2067085 C allele and concomitant splicing to exon 3**. Sequencing of RT-PCR amplicons from human leukocyte cDNA to elucidate the processing of the primary transcript in the regions of exons 2-3a and 3a-4 as well as 2-3 and 3-4. Fluorescence traces are given for the SNP context in exon 2 (rs2067085, minor allele frequency 0.388), exon 3a (rs34133110) and exon 4 (rs2066842, minor allele frequency 0.362). Line: primary transcript; boxes: exons, arrows: PCR primers; dotted lines: amplifiable region; angulated lines: observed splice events.

**Figure 4:**
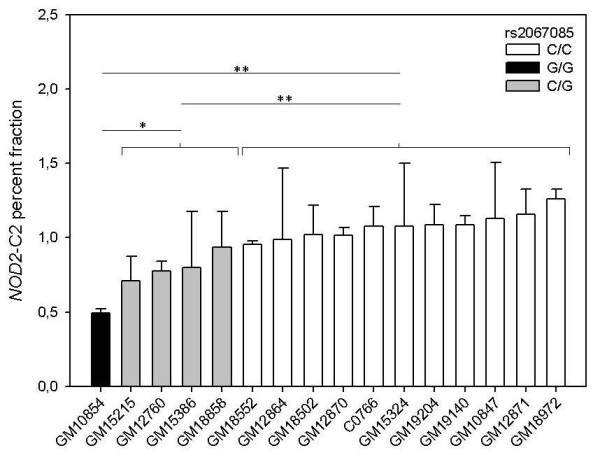
**rs2067085 genotype-specific expression of the *NOD2*-C2 transcript in human LCLs**. The expression rate was determined by RT-PCR spanning *NOD2 *exon 2-4 and calculated as percent fraction of exon 3a inclusion vs. all processed transcripts from this region. Values represent the mean ± standard deviation of triplicate RNA isolations. Statistical significance (WMRST): * p < 0.05; ** p < 0.01.

It has been shown that short NOD2 isoforms exhibit divergent functions compared to NOD2. This includes either NOD2-S [22] or its mutated forms associated with susceptibility for Crohn's disease [15,16] as well as artificial ones consisting of both CARD domains [24]. So far, no evidence for endogenous *NOD2 *transcript isoforms encoding for a truncated CARD-CARD protein existed. Studying human lymphoblastoid cell lines (LCLs), we identified a respective alternative transcript, called *NOD2*-C2. It results from inclusion of a - so far unknown - exon between the exons 3 and 4 (termed 3a; genomic coordinates: hg19, chr16:49,301,356-49,301,428; Figure 1). Inclusion of exon 3a adds nine amino acids (aas, 189-197) to the C-terminal part of the encoded NOD2-C2 that differ to those at the respective NOD2 position (see Additional file 1). For complementation of the second CARD only the aa positions 188-193 are of relevance [24]. Although the respective aas are altered in NOD2-C2, prediction of the secondary protein structure [26] suggests a helix motif for the aas 182-197. The same motif has been predicted for the related aas of NOD2 (208-227 aa). Therefore it is likely, that complementation of NOD2-C2 s second CARD by inclusion of exon 3a creates a domain with NOD2-like functional capability. From a genetic point of view, exon 3a resides in an interspersed MIRb repetitive element. Both acceptor and donor are identical to the consensi of other exonized ALUs and MIRs, respectively [27,28]. The sequence of exon 3a is 100% conserved in chimpanzee and *NOD2*-C2 transcripts are also detectable in a chimpanzee cell line (data not shown). Exon 3a containing transcripts are predominantly expressed in leukocytes as well as in other tissues like spleen, testis or placenta (Figure 2). Exon 3a inclusion is coupled to the concomitant inclusion of exon 3 and transcription initiation at exon 1a (nomenclature according to [29]). Moreover, we screened human tissue cDNA panels for allelic imbalance in *NOD2*-C2 transcription and detected predominant expression from the major allele of the synonymous coding SNP rs2067085 (C, Ser 178) in exon 2 (Figure 3). Since *NOD2*-C2 expression is coupled to exon 3a splicing other potentially splice-regulatory SNPs in the coding and flanking regions of exon 3a were checked. However, neither rs34133110 (exon 3a) nor rs2066842 (exon 4) showed signs of allelic imbalance. Moreover, since exon 3a inclusion requires concomitant inclusion of exon 3, we examined rs2067085 (exon 2) and rs2066842 (exon 4) for association with exon 2-3-4 splicing but found no correlation. Due to the unknown rs2067085 allele frequencies (HapMaps CEU: C/G 0.612/0.388) of the used cDNA tissue panels, expression of minor alleles may be underestimated by sequencing. Therefore, we screened 14 human LCLs for rs2067085 genotype-specific differences in *NOD2*-C2 transcription by quantitative RT-PCR spanning the exons 2-4 (Figure 4). Among the LCLs we identified 11/1 homozygous for C and G, respectively, as well as 4 heterozygous cell lines. Comparing all three rs2067085 genotypes, significant differences in *NOD2*-C2 transcript expression were observed. The homozygous G LCL showed the lowest relative expression (0.49 ± 0.03%), whereas expression is doubled in homozygous C LCLs (1.08 ± 0.09%; p = 0.005, WMRST). Heterozygous LCLs had an intermediate (0.81 ± 0.10%) expression significantly different from those of homozygous G (p = 0.012, WMRST) and C (p = 0.001, WMRST) individuals. Our results identified *NOD2*-C2 transcripts being 2-fold higher expressed from the major allele of rs2067085 compared to the minor one (Figure 4).

### NOD2-C2 activates NF-κB in an MDP independent manner

**Figure 5:**
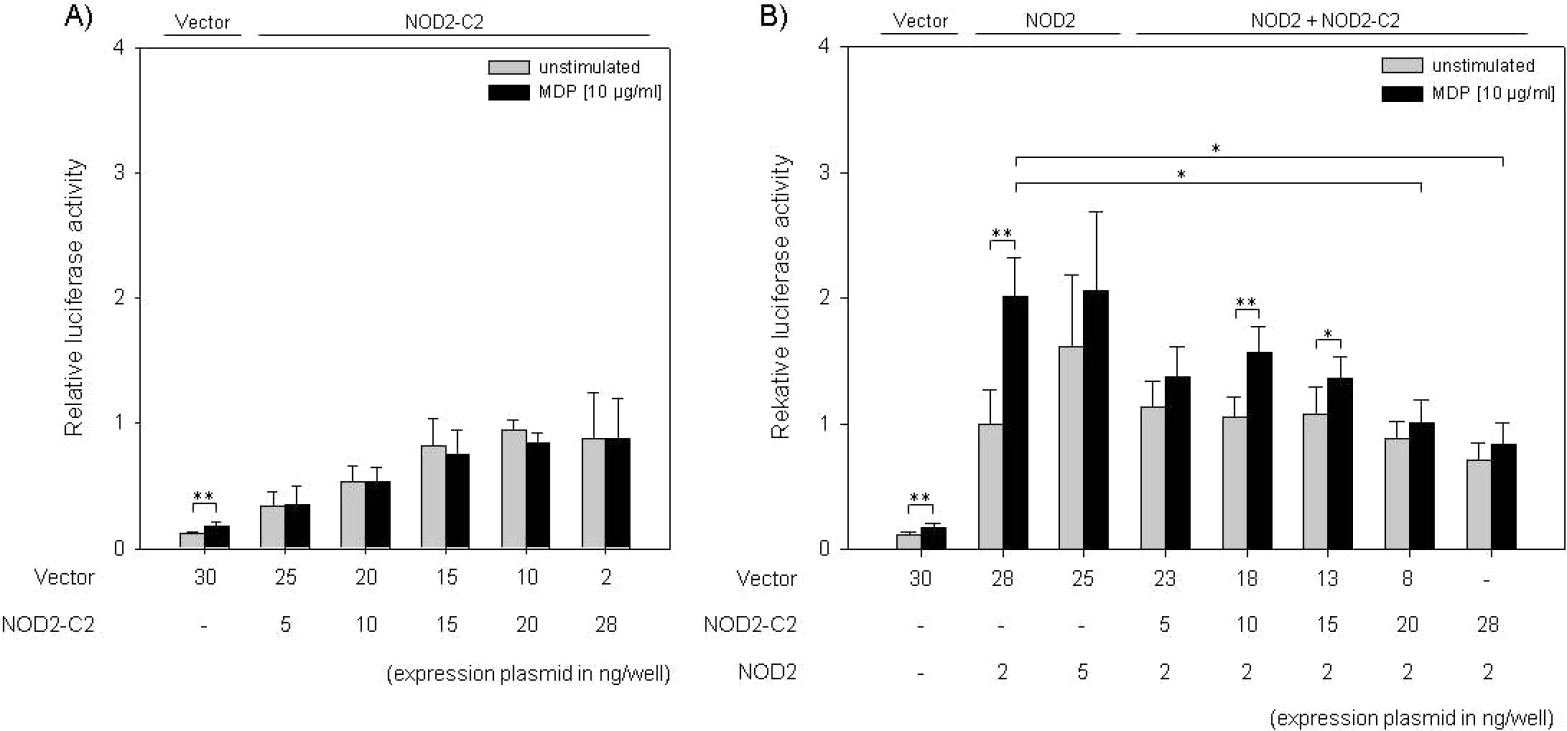
**MDP-independent activation of NF-κB by NOD2-C2**. Relative NF-κB activity was measured using a luciferase-based NF-κB reporter assay. HEK293 cells were cotransfected with NOD2-C2 (A), NOD2 or NOD2-C2/NOD2 (B) expression constructs and dual-lucifease reporter plasmid. 10 μg/ml MDP were used to stimulate NOD2-mediated NF-κB activation. Relative luciferase activity (compared to NOD2 (2ng) without MDP stimulation) is plotted against NOD2 and/or NOD2-C2 plasmid amounts. Values represent the mean ± standard deviation of triplicate cultures. Statistically significant differences are marked by asterisk * p < 0.05; ** p < 0.01.

The ability of NOD2-C2 for activation of NF-κB and subsequent downstream signaling was analyzed using a dual-luciferase reporter assay system. HEK293 cells expressing only very low levels of endogenous NOD2 [10,30] had been transfected with increasing amounts (0-28 ng) of NOD2 or NOD2-C2 expression construct. All experiments were additionally carried out in cells stimulated with MDP (10 μg/ml), an activator of the NOD2-dependent NF-κB activity (Figure 5, black bars). All values are given as relative luciferase activity compared to the NOD2 construct (2 ng) without MDP stimulation (Figure 5B). NOD2-C2 is able to stimulate NF-κB activity in a dose dependent manner (p < 0.05; Tukey Test; Figure 5A). The presence of MDP did not affect the ability of NOD2-C2 to activate NF-κB (Figure 5A). As a control, NOD2 also activates NF-κB (Figure 5B). In contrast to NOD2-C2 this activation is not dose sensitive, rather turns into a steady-state level. To evaluate the NOD2-dependent activation of NF-κB by NOD2-C2 a titration of the NOD2-C2 construct against constant amounts of NOD2 (2 ng) expression vector was performed. The activity of NF-κB remains constant independently of the NOD2-C2 plasmid amounts (Figure 5B). At the same time, NOD2-C2 abolishes the stimulatory effect of MDP onto NOD2-induced NF-κB activation (2-fold; p = 0.001 for 0 ng NOD2-C2 construct) and leads to an even decreased NF-κB activity in a dose-dependent manner (p < 0.05 for 20 ng and 28 ng NOD2-C2 construct; Figure 5B).

### NOD2-C2 is localized in the cytoplasm

**Figure 6:**
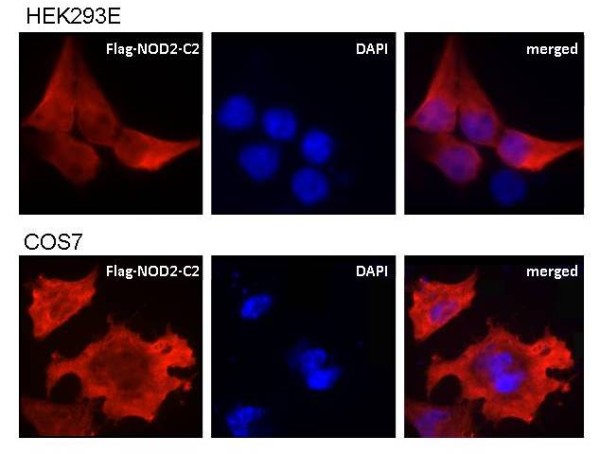
**Cytosolic localization of NOD2-C2**. COS7 and HEK293E cell were transiently transfected with Flag-tagged NOD2-C2 construct and after 24 h immunostained with anti-Flag antibody.

The cellular localization of NOD2-C2 in african green monkey kidney COS7 and human embryonic kidney HEK293E cells was carried out by immuno-fluorescence confocal-like microscopy. 24 h after transfection NOD2-C2 was cytosol-localized (Figure 6). We could not detect a general localization of NOD2-C2 at other cellular compartments than the cytoplasm.

## Discussion

Several studies on NOD2 paid attention to its functional domains by creating truncated isoforms and found remarkable differences in their potential to activate NF-κB compared to wild type [24,30]. Beside transcripts carrying CD-associated polymorphisms (Arg702Trp, Gly908Arg and Leu1007sinsC) [15-18] only the alternative splice isoform *NOD2*-S and LRR-modifying exon skipping have been found to be *in vivo *detectable variations [15-18,22,23] with a potential regulatory input in NOD2 signaling. This is surprising, since Leung and colleagues found a large set of alternative splicing events in a detailed analysis of *NOD2 *transcripts [23]. As far as variations in the NOD or LRR domains are almost loss of function events, it turned out that the number of CARDs in CARD-only isoforms like NOD2-S seems to be critical for acting as positive or negative regulators of CARD-dependent caspase- or NF-κB-signaling pathways.

Our analysis revealed a novel *NOD2 *alternative transcript, *NOD2*-C2, coding for a unique tandem CARD feature. We observed that *in vitro *(i) NOD2-C2 alone interferes with the activation of NF-κB in a dose dependent manner, which is quite in contrast to NOD2-S that inhibits NF-κB activation, and (ii) NOD2-C2 in combination with NOD2 abolishes the stimulatory effect of MDP. The first finding is in agreement with an initial study by Ogura *et al. *in which it was shown that both CARDs of NOD2 are essential for RIP2 recruitment during induced proximity signaling and that neither the first nor the second CARD alone are able to interact with RIP2 [24]. This unique feature of the NOD2/RIP2 interaction has not yet been fully resolved on the structural level, because other RIP2-interacting proteins, possess only a single CARD domain (e.g. NOD1, bcl10, and caspase-1). The ability of NOD2-S to bind RIP2 sheds further light on the complexity of this interaction, because NOD2-S exhibits only 54 out of 93 amino acids of NOD2s second CARD. Nevertheless, this is sufficient to mediate the attachment of RIP2, but not the subsequent NF-κB activation. Thus the NOD2-S/RIP2 binding results in RIP2 inhibition [22]. Critical amino acids for the NOD2/RIP2 binding have been found in both CARDs [30] suggesting a activation of RIP2 by those part of the second CARD which are truncated in NOD2-S. There is a similar example for a tandem CARD signaling molecule. The retinoic acid-inducible gene-I protein (RIG-I) recognizes viral RNA and initiates an antiviral signaling cascade [31]. Upon viral infection both N-terminal RIG-I CARDs are needed for proper interaction with downstream signaling partners like MAVS or Cardif. Interestingly, while the first CARD is essential for binding of TRIM25 (tripartite motif protein 25) the second one enables TRIM25-mediated RIG-I ubiquitinylation [31]. Since NOD2 signaling requires the interaction with the kinase RIP2, further analyses should be addressed to potential CARD post-translational modifications like phosphorylation using NOD2-C2 as biologically relevant experimental systems. In line with NOD2-S, a RIG-I splice variant with a short deletion in the first CARD acts as an endogenous inhibitor of RIG-I signal transduction, possibly providing a negative-feedback inhibition or fine-tuning mechanism [31].

Whatever the NOD2-C2 functions *in vivo *explicitly are, our *in vitro *findings point to a sub-functionalization of NOD2 isoforms in innate immunity. Similar to NOD2-S, NOD2-C2 abolishes the stimulatory effect of MDP, but does not significantly decrease NOD2-mediated NF-κB activity in the absence of MDP. We assume a NOD2-C2/NOD2 interaction preventing the NOD2 self oligomerization that followed MDP stimulation and subsequent RIP2 activation as it has been described for NOD2-S [22]. This inhibition is compensated by the ability of NOD2-C2 to activate NF-κB in a MDP-independent manner. Since NOD2-C2 shares the vast majority (163) of its 205 amino acids with NOD2-S, similar to NOD2-S/NOD2 a potential for NOD2-C2/NOD2 interaction exists. Additionally, we observed NOD2-C2 cytosolic localization as it has been described for NOD2. Although NOD2 may also be associated with the plasma membrane [10,12-14], this is not expected for NOD2-C2 due to the absence the COOH-terminal domain of NOD2 required for membrane association [10]. Nevertheless, a potential interaction of NOD2-C2 with other membrane-associated molecules like CD147 or Erbin has already been assumed, since both NOD2 CARDs are needed for sufficient interaction. CD147 is a regulator of cellular migration, differentiation and inflammatory processes [14] and Erbin potentially regulates NOD2 and is involved in cell polarity, receptor localization and regulation of MAPK pathways [32]. This suggests that NOD2-C2 features a multi-facetted interaction potential and to date unknown functional spectrum. *NOD2*-C2 transcripts are predominantly expressed in leukoyctes, cells of the immune system defending the host against both infections and pathogen materials. Together with NOD2-C2s ability to activate NF-κB in a MDP-independent manner we assume that NOD2-C2 acts as an endogenous inductor of a basal immune response to handicap pathogen invasion.

Expression levels of *NOD2*-C2 transcripts are low but consistent in human tissues and LCLs (about 1% of all NOD2 transcripts when inspecting exons 2-4 by quantitative RT-PCR). Nevertheless, overexpression of an artificial NOD2 tandem CARD construct was toxic for HEK293T cells [32]. This agrees with the low amounts of *NOD2*-C2 transcripts in LCLs. It is plausible that NOD pathways could turn on apoptosis pathways (such as caspase induction), but the final outcome of cell death would be generally masked by the overriding anti-apoptotic power of NOD-mediated activation of NF-κB pathways. Nevertheless, it was shown that Nod1-dependent apoptosis is a caspase 8-mediated event and that apoptosis requires RIP2 [33]. Therefore, further studies have to be performed to reveal NOD2-C2s involvement in cell death. Interestingly, the expression of the *NOD2*-C2 transcript in LCLs was significantly different for all three possible rs2076085 genotypes with 2-fold higher expression of the C allele compared to G. The observed allele-preferential expression of *NOD2*-C2 transcripts allows speculation about potential implications with respect to inflammatory abnormalities like CD. So far, genome-wide association studies did not uncover rs2067085 being associated with CD or ulcerative colitis (UC). Juval and colleagues found that rs2067085 allele and genotype frequencies did not differ between controls and UC patients [34]. Nevertheless, homozygous G individuals are not common in HapMaps [35] CEU (Utah residents with Northern and Western European ancestry from the CEPH collection) population (17,2%) and are under-represented in their Caucasian cohorts (with 8% in controls and 6,3% in UC patients). Interestingly, this genotype is rare in Africans YRI (YRI - Yoruba in Ibadan, Nigeria, 3.4%) and totally missing in Asians (JPT - Han Chinese in Beijing, China and HCB - Japanese in Tokyo, Japan). Thus, studies on a larger population of European ancestry may reveal an association of the minor rs2067085 allele and disease driven by chronic inflammation.

The mechanistic impact on splicing of the synonymous SNP rs2067085 (C/G, Ser/Ser) is not known. Its localization 7 nucleotides upstream of the exon/intron boundary let us speculate about potential splice regulatory functions [36]. Although the ESE finder predicts different splicing factor binding sites for both SNP alleles [31], we found no association with the inclusion or skipping of the downstream exon 3. On the other hand, exon 3a inclusion is only observed in transcripts carrying exon 3. Although exon 3a is in the primary transcript 10 kb downstream of exon 2, it derives twice as often from the transcribed C in comparison to the G allele of rs2067085. It may well be that inclusion of exon 3a depends on an upstream splicing event as it has been reported for the thrombopoietin gene where the recognition of the donor and acceptor sites of an intron requires spliceosome assembly on upstream splice sites [37].

Similarly, efficient splicing of *NF1 *is guided by distant sequence features [38]. No reports that could explain our finding are available. Thus, detailed studies, for instance using minigene constructs, may provide further insights into NOD2 splicing. Linkage to other SNPs potentially affecting gene expression, like promoter SNPs, has not been tested. Nevertheless we have shown by 5'RACE that *NOD2*-C2 exclusively contains the recently identified exon 1a [29] and is therefore transcribed from the alternative NOD2 promoter, whereas transcription of the canonical exon 1 is regulated by an NF-κB-dependent promoter. This may lead to tissue-specific and context-dependent *NOD2 *transcript isoform patterns [29], with respect to independently regulated expression of *NOD2 *and *NOD2*-C2 transcripts in the context of inflammatory signaling. Although there is a low basal NOD2 expression in chimpanzee LCLs, we found *NOD2*-C2 transcripts being expressed too (data not shown). This is surprising since a number of genes involved in the inflammatory response - *APOL1*, *APOL4*, *CARD18*, *IL1F7*, *IL1F8 *- are entirely deleted from chimp genome [39]. In humans, *APOL1 *is involved in resistance to the parasite that causes sleeping sickness [40], while *IL1F7 *and *CARD18 *play a role in regulating inflammation. Therefore, there has to be a different regulations of these processes in chimpanzees [41-43]. In this context the existence of *NOD2*-C2 transcripts at low levels similar to the situation in humans supports the assumed importance in basal NF-κB activation.

Due to the lack of suitable antibodies for C-terminal truncated NOD2 isoforms, we could not detect endogenous NOD2-C2 *in vivo*. In particular, we obtained irresolvable multiple bands employing Western blotting with commercial antibodies raised against N-terminal peptides. Formally, the *NOD2-*C2 transcript isoform appears as a target for nonsense-mediated mRNA decay (NMD) as it harbors a premature stop codon within exon 3a followed by at least one exon/exon junction [44]. The exceptionally long fourth exon (1.8 kb) hampers the analysis of downstream transcript structure, but increases the possibility to overcome NMD e.g. by alternative polyadenylation sites. Furthermore, the consistent transcript levels and the evidence for NOD2-S protein translation [22] indicate that NMD or other mechanisms in transcripts with premature stop codons (e.g. negative regulation of nuclear export or translation) may not play a functional role for *NOD2*-C2 transcripts [45]. Interestingly, corresponding truncated variants of R genes in plants also seem to escape NMD [46]. Thus, future studies have to elucidate whether *NOD2*-C2/-S transcript isoforms belong to the class of NMD escapers.

## Conclusions

NOD2 transcripts carrying an alternative exon downstream of exon 3 (exon 3a) are the endogenous equivalents of a previously described *in vitro *construct with the putative protein composed of only the two N-terminal CARDs. This protein form (NOD2-C2) activates NF-κB independent of an MDP stimulus and is a potential regulator of NOD2 signaling.

## Competing interests

The authors declare that they have no competing interests.

## Authors' contributions

MK, PR, MP, CK, SS and KH designed the study. MK, JB, DR performed the laboratory work. MK, JB, PR, MP, CK, SS and KH performed data analysis. Interpretation of data and writing of the manuscript were done by MK, PR, MP, CK, SS and KH. All authors read and approved the manuscript.

## Supplementary Material

Additional file 1 **Amino acid alignment of NOD2 and NOD2-C2**. ClustalW output for the amino acid sequences of NOD2-C2 and partial of NOD2 (full length 1040 aa). NOD2 CARD domains as assessed by Ogura *et al. *[24] are highlighted by grey boxes. Amino acid conservation is indicated by the following symbols: (*) single, fully conserved residue, (:) conservation of strong groups, (.) conservation of weak groups and (-) no consensus. Click here for file
