# Supplementary material for: NOD2-C2 - a novel NOD2 isoform activating NF-κB in a muramyl dipeptide-independent manner
Source: BMC Res Notes. 2010 Aug 10;3:224. doi: 10.1186/1756-0500-3-224 (PMC2931527; doi:10.1186/1756-0500-3-224)
Supplement: Additional file 1 — Amino acid alignment of NOD2 and NOD2-C2. ClustalW output for the amino acid sequences of NOD2-C2 and partial of NOD2 (full length 1040 aa). NOD2 CARD domains as assessed by Ogura et al. [24] are highlighted by grey boxes. Amino acid conservation is indicated by the following symbols: (*) single, fully conserved residue, (:) conservation of strong groups, (.) conservation of weak groups and (-) no consensus. [file 1756-0500-3-224-S1.PDF]

```
                                   CARD
NOD2       MGEEGGSASHDEEERASVLLGHSPGCEMCSQEAFQAQRSQLVELLVSGSLEGFESVLDWL   1-60
NOD2-C2    ---------------------------MCSQEAFQAQRSQLVELLVSGSLEGFESVLDWL   1-33
                                      *********************************

NOD2       LSWEVLSWEDYEGFHLLGQPLSHLARRLLDTVWNKGTWACQKLIAAAQEAQADSQSPKLH   61-120
NOD2-C2    LSWEVLSWEDYEGFHLLGQPLSHLARRLLDTVWNKGTWACQKLIAAAQEAQADSQSPKLH   34-93
           ************************************************************

                 CARD
NOD2       GCWDPHSLHPARDLQSHRPAIVRRLHSHVENMLDLAWERGFVSQYECDEIRLPIFTPSQR   121-180
NOD2-C2    GCWDPHSLHPARDLQSHRPAIVRRLHSHVENMLDLAWERGFVSQYECDEIRLPIFTPSQR   94-153
           ************************************************************

NOD2       ARRLLDLATVKANGLAAFLLQHVQELPVPLALPLEAATCKKYMAKLRTTVSAQSRFLSTY   181-240
NOD2-C2    ARRLLDLATVKANGLAAFLLQHVQELPVPLALPLEDERTEAQKG----------------   154-197
           ***********************************    :   .
```
